# Acute Right Ventricular Dysfunction in Intensive Care Unit

**DOI:** 10.1155/2017/8217105

**Published:** 2017-10-19

**Authors:** Juan C. Grignola, Enric Domingo

**Affiliations:** ^1^Pathophysiology Department, School of Medicine, Hospital de Clínicas, Universidad de la República, Montevideo, Uruguay; ^2^Postoperative Cardiac Critical Care Unit, Centro Cardiológico Americano, Montevideo, Uruguay; ^3^Area del Cor, Hospital Vall d'Hebron, Barcelona, Spain; ^4^Physiology Department, School of Medicine, Universitat Autonoma de Barcelona, Barcelona, Spain

## Abstract

The role of the left ventricle in ICU patients with circulatory shock has long been considered. However, acute right ventricle (RV) dysfunction causes and aggravates many common critical diseases (acute respiratory distress syndrome, pulmonary embolism, acute myocardial infarction, and postoperative cardiac surgery). Several supportive therapies, including mechanical ventilation and fluid management, can make RV dysfunction worse, potentially exacerbating shock. We briefly review the epidemiology, pathophysiology, diagnosis, and recommendations to guide management of acute RV dysfunction in ICU patients. Our aim is to clarify the complex effects of mechanical ventilation, fluid therapy, vasoactive drug infusions, and other therapies to resuscitate the critical patient optimally.

## 1. Introduction

The role of the left ventricle (LV) in ICU patients with circulatory shock has long been considered. However, acute right ventricle (RV) dysfunction causes and exacerbates many common critical illnesses (e.g., acute respiratory distress syndrome (ARDS), pulmonary embolism (PE), inferior acute myocardial infarction, and postoperative cardiac surgery).

There is a variety of definitions for acute RV dysfunction (RVD), RV failure (RVF), and right heart failure (RHF) in the literature that must be clarified and not used interchangeably.

*RHF* can be defined by a clinical syndrome due to an alteration of structure and/or function of the right heart circulatory system (comprised from the systemic veins up to the pulmonary capillaries) that reduces the ability to propel blood to the pulmonary circuit and/or high systemic venous pressures at rest or with effort [1]. Failure of the RV is a frequent component of RHF but not a mandatory feature of the RHF syndrome.

*Acute RVD* is defined as at least one of the following (Table 1) [2, 3]:

- Acute occurrence of RV systolic dysfunction by measuring the longitudinal systolic displacement and dilation [4–6]
- Unexplained increase of natriuretic peptides in the absence of LV or renal disease
- Electrocardiographic (ECG) RV strain patterns which are strong markers of moderate-to-severe RV strain. While specific, they are limited by a lack of sensitivity.

Evidence of cardiomyocyte death (elevation of troponin *I* > 0.4 ng/mL, troponin *T* > 0.1 ng/mL) predicts severe RVD. Although evidence of cardiomyocyte death can be seen in the absence of RVD, such patients are at risk for progression to circulatory collapse.

*Acute cor pulmonale* (ACP) is a form of RVD due to an acute increase in RV afterload.

**Figure 1:**
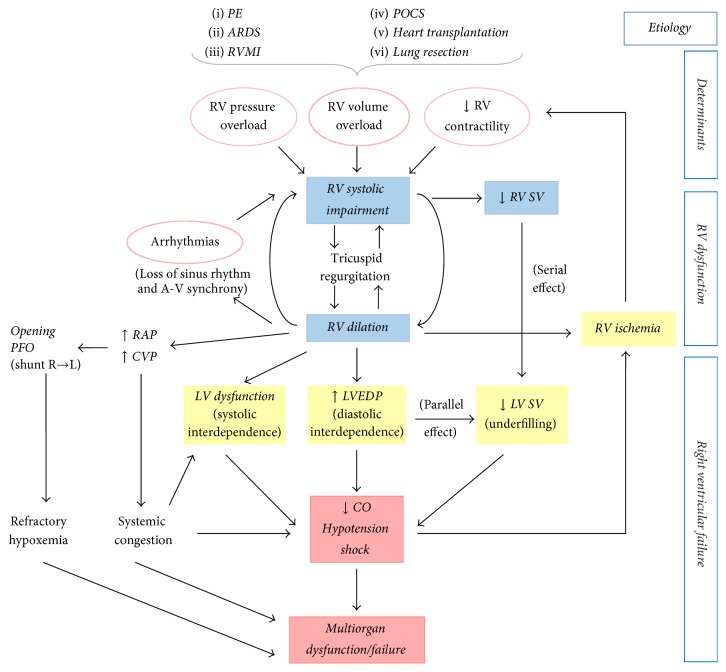
Mechanisms of acute right ventricular dysfunction/failure (RVD/RVF). RV dysfunction begins with excessive increases in preload or afterload or injury that results in decreased contractility. RV ischemia and LV function impairment ensue a vicious cycle worsening hemodynamics and precipitate the transition to RVF. ARDS: acute respiratory distress syndrome; A-V: atrioventricular; CO: cardiac output; CVP: central venous pressure; MI: myocardial infarction; PE: pulmonary embolism; PFO: patent foramen oval; POCS: postoperative cardiac surgery; RAP: right atrial pressure; R → L: right-to-left; SV: stroke volume.

*Acute RVF* is defined as acute RVD plus low cardiac output (CO) and hypoperfusion with the consequent multiorgan dysfunction/failure. RVF occurs when the RV fails to provide enough blood flow to the pulmonary circulation to accomplish adequate LV filling [7] (Figure 1). It can be suspected whenever the ratio of the right atrial pressure to the pulmonary arterial occlusion pressure ≥ 0.8–1.0 with a reduction in the cardiac index.

In the present work, we will focus on the epidemiology, pathophysiology, diagnosis, and treatment of acute RVD/RVF.

## 2. Epidemiology

Acute RVD is both common and potentially lethal in critically ill patients. Different clinical entities can produce acute RVF in ICU as a consequence of alterations in one or more of the determinants of RV performance (preload, afterload, and contractility). We will discuss the clinically most important etiologies of acute RVD/RVF:

- Acute PE is a common cause of acute RVD/RVF due to an excessive increase in afterload secondary to obstruction by clots, vasoconstriction in nonobstructed areas, and intracardiac hemolysis (resulting from the turbulent flow across the pulmonary value). Echocardiographic RVD is present between 30 and 56% of normotensive patients with PE. All-cause mortality rate at 30 days in the patients with confirmed PE was 5.4 to 10%, and in-hospital mortality rate directly attributed to PE was 1.1 to 3.3%, depending on whether it is in-patients versus out-patients registry and the degree of illness [8–11]. Cardiogenic shock occurs in ~5% of acute PE cases with a 90-day mortality rate of more than 50% [12]. In general, in previously healthy and nonremodeled RV-pulmonary unit at least 40% of the cross-sectional area must be obstructed to significantly increase the pulmonary arterial pressure (PAP), and besides RV cannot acutely overcome a systolic PAP more than 50 mmHg [13]. Conversely, acute-on-chronic RVF can tolerate significantly higher PAP [14].
- ARDS is one of the most common entities to challenge the RV. The incidence of acute RVD in ARDS varies from 30 to 56%, depending on the definition criteria of RVD, the severity of lung injury, and ventilatory strategy which is associated with increased 28-day mortality even in the lung-protective mechanical ventilation era and Berlin definition of ARDS [15–17]. Both pulmonary hypertension and RV contractile impairment are the main factors involved in RVD [18, 19]. Mechanisms of ARDS-induced acute RVD include hypoxic/hypercarbic vasoconstriction, an increased alveolar dead space, pulmonary microthrombi, and proinflammatory cytokine activation. A recent study identified four predictors of acute RVD in ARDS: (1) pneumonia-induced ARDS, (2) partial pressure of arterial oxygen/fraction of inspired oxygen ratio < 150 mmHg, (3) partial pressure of carbon dioxide ≥ 48 mmHg, and (4) driving pressure (plateau pressure − total positive end-expiratory pressure) ≥ 18 cmH_2_O [17]. Routine echocardiography is recommended in all ARDS patients with a score ≥ 2 (incidence of RVD ≥ 20%) allowing an early implementation of RV-protective strategy that might prevent RVD.
- RV myocardial infarction (RVMI) can be complicated by acute RVD in 30–50% of patients with inferior wall ST-elevation MI. Meanwhile, severe hypotension and low CO are present in 10% on admission in the reperfusion era [19]. The right coronary artery (RCA) usually is the culprit vessel in RVMI, and more extensive RV myocardial necrosis is associated with proximal RCA occlusions [20]. The RV tolerates ischemic injury better than the LV because it has a lower oxygen demand, greater coronary flow reserve, dual, right and left, coronary arteries supply, and homogeneous transmural perfusion across the cardiac cycle [21]. Although RVMI increases the risk of complications in patients with inferior MI, several studies have reported that the acute outcome of patients with RVMI is primarily determined by the amount of accompanying LV necrosis [22].
- Acute RVF is a serious problem after cardiothoracic surgery. It occurs in 0.1% of patients after cardiotomy, in 2-3% of patients undergoing heart transplantation, and in 10–20% of patients needing LV assist device insertion [23]. PH and myocardial depression after cardiopulmonary bypass are usually mild, except in vulnerable patients, to whom it may contribute to postoperative RVF. In the postoperative cardiac surgery (POCS) patient, acute RVD (RV fractional area change ≤ 25% or severe RV dilation) was present in almost half of the patients hemodynamically unstable. Several factors may be implicated to RVD/RVF in the POCS patient: (a) long cardiopulmonary bypass time, (b) right coronary embolism or bypass graft occlusion, (c) inadequate myocardial protection during surgery, (d) reperfusion lung injury with secondary PH, (e) protamine-induced pulmonary hypertension (PH), (f) atrial arrhythmias or loss of atrioventricular synchrony, and (g) preexisting pulmonary vascular disease [24, 25].
- The extent of pulmonary parenchymal resection (loss of pulmonary tissue) and the preexisting PVD/RVD predict the risk and severity of postoperative RVD in patients undergoing lung resection. Hypoxia, atelectasis, and hypercarbia may precipitate acute RVD [26].

## 3. Pathophysiology of Acute RV Dysfunction and Failure

### 3.1. Anatomy and Mechanics of RV

The anatomy and physiology of the RV are both unique and complex and quite different from LV. In contrast to the ellipsoidal shape of the LV, the RV appears triangular and crescent-shaped. Anatomically, RV can be described regarding three components: (1) the inlet, which consists of the tricuspid valve, chordal tendineae, and papillary muscles; (2) the trabeculated apical myocardium; and (3) the infundibulum, or conus, which corresponds to the outlet region [27]. Data from phylogeny suggest that the infundibulum can be found as early as in primitive chordates and the RV sinus is found quite later in vertebrates, presumably as an adaptation of the cardiovascular system to air breathing. In crocodiles, venous and arterial circulation diverged for the first time, with an infundibulum incorporated to the RV. In birds and mammals, this incorporation is complete [27, 28]. According to which ontogeny reflects the phylogeny, the infundibulum is present in very early stages of mammalian embryonic development (20 days after fecundation), while the RV sinus develops later (approximately 22 to 24 days after fecundation) [29].

Regarding the myofiber architecture of the heart and according to Torrent-Guasp and other authors, the ventricular myocardium is constituted by a continuous band of muscle that extends from the pulmonary artery root to the aortic root, forming a helical structure with two spirals and delimiting the two ventricular cavities. This myocardial band would be composed of the “basal loop” and the “apical loop.” The basal loop is predominantly horizontal and comprises the right and left segments; the apical loop is predominantly vertical and consists of the descending segment (“left septum”) and the ascending segment (“right septum”) [32–35].

Under normal afterload, RV contraction begins at the sinus (inlet chamber) and progresses toward the conus or infundibulum (outlet chamber) (approximately 25 to 50 ms apart), indicating a peristaltic/asynchronous bellows-like pattern of contraction from apex to base. In contrast, LV contracts in a squeezing/synchronous pattern by twisting and rotational movements from apex to base (likened to wringing a towel) [36]. The RV contracts by three mechanisms: (1) inward movement of the free wall secondary to the contraction of the right segment of the basal loop (transverse orientation), which produces a bellows effect; (2) contraction of the ascending segment of the apical loop (oblique orientation), which shortens the long axis, drawing the tricuspid annulus toward the apex; and (3) traction on the free wall at the points of attachment secondary to LV contraction [37–39]. The shortening of the RV is mainly longitudinal compared to radial, and the sinus chamber made up 81 ± 6% of the RV end-diastolic volume and 87 ± 4% of the stroke volume [36].

The low impedance and the high capacitance of the normal pulmonary circulation are reflected in the triangular shape of the RV pressure-volume loop, without distinct periods of isovolumic contraction and relaxation [40–43]. RV ejection begins early during the increase of intraventricular pressure and continues during its fall. This prolonged low-pressure ejection implies that RV emptying is very sensitive to changes in afterload and that RV keeps on ejecting (late phase of ejection) while the LV is in diastole (isovolumic relaxation and rapid filling phases or presuction and suction phases, resp.). It corresponds to the contraction of the ascending segment of the apical loop without opposition of the descending segment that is relaxed (named by Torrent-Guasp “late isovolumetric contraction”) [38].

### 3.2. Pathogenesis of Acute RV Dysfunction and Failure

RV mechanics and function can be altered in the setting of either pressure/volume overload and primary reduction of contractility owing to myocardial ischemia (Figure 1). The compliant and thin walled RV is better suited to accommodate significant increases in preload but tolerates acute increases in afterload poorly.

The heart has intrinsic mechanisms to maintain CO to beat-to-beat changes in preload and afterload by a heterometric dimension adaptation described by Starling's law of the heart. Myocardial stretch elicits a rapid increase in developed force, which is mainly caused by an increase in myofilament calcium sensitivity (Frank-Starling mechanism). In the next 10–15 min, a second gradual increase in force takes place (slow force response), increasing the calcium transient amplitude secondary to a cardiac autocrine-paracrine nongenomic mechanism and named homeometric autoregulation described by Von Anrep more than 100 years ago [44]. Although this homeometric adaptation to afterload has been demonstrated in the RV exposed to pulmonary arterial constriction, RV stroke volume falls sharply beyond mean PAP of 30 mmHg [45]. Our group, working with anesthetized, opened pericardium sheep, observed that the asynchronous and sequential RV contraction with normal afterload changed to a synchronic contraction pattern during acute and moderate PH. RV contraction synchronization allowed RV to increase contractility, keeping both CO and end-diastolic volume constant [46]. In another experimental model of a stepwise increased pulmonary arterial pressure, we showed that the RV could initially (systolic PAP of 30 mmHg) improve its systolic function through an homeometric autoregulation mechanism. When systolic PAP reached 35 mmHg the systolic performance increase was lost, returning to the baseline value and the active diastolic function was impaired without either dilation or significant changes in ventricular compliance. Acute RVF and circulatory collapse came at a systolic PAP > 40 mmHg [47].

Acute adaptation of the RV to PH depends on both the stationary (pulmonary vascular resistance) and the pulsatile (PA stiffness, total pulmonary capacitance, and reflected wave) components of afterload [48]. It should be considered that the dynamic afterload may be different according to the clinical scenarios. We have shown that, during active PH (phenylephrine induced vasoconstriction), the RV pulsatile load was attenuated through preserving proximal PA stiffness and total pulmonary capacitance and decreasing the magnitude of the reflected wave in comparison with isobaric PA banding [49]. Both the PE and the increase of the mPAP secondary to the increase in the left atrial pressure would determine a predominant increase of the pulsatile load unlike the ARDS with an effect preferably on the stationary load [50–53]; therefore the former could present circulatory collapse at a lower mPAP.

RV systolic impairment and dilation emerge once both myocardial intrinsic adapting mechanisms are exhausted. Several molecular and cellular mechanisms have been proposed in the development of acute RVD secondary to PH. RV wall tension increase leads to the cardiomyocyte stress and injury secondary to ischemia, substrate depletion, and mitochondrial energy metabolism impairment [54]. Different amplifying loops have been involved in the contractile dysfunction, enforcing further stress on the remaining cardiomyocytes. Among them, neutrophil-mediated inflammation secondary to the influx of proinflammatory cells and chemokine/cytokine activation play the main role by producing oxidative damage, cardiomyocyte apoptosis, and direct negative inotropic effects (myosin heavy chain switch and the decrease of myofibrillar sensitivity to calcium). All of them state a proinflammatory phenotype of RV [54–57].

The biochemical and mechanical changes accounting for the transition from acute RVD to failure remain a subject of intense study. Some authors have proposed that acute RV failure begins when the coronary vasodilator reserve is exhausted as a consequence of RV ischemia although it is not possible to discard the concomitant existence of a primary RV failure, related to an overdistension of the ventricle [58, 59]. Another mechanism proposed is LV mechanical dysfunction by ischemia and edema, which can lead to RVD through systolic and diastolic ventricular interdependence [60, 61]. The upstream transmission of LV end-diastolic pressure to left atrial pressure, pulmonary arterial wedge pressure, and mean PAP may approach a 1 : 1 ratio, producing a vicious cycle.

Finally, RV cardiomyocyte ischemia produces another vicious cycle of increased oxygen demand in the setting of decreased oxygen delivery, leading to circulatory collapse and multiorgan failure (Figure 1).

## 4. Clinical Presentation and Diagnosis of RVD/RVF in ICU

The clinical presentation of acute RVF varies depending on the underlying cause, the presence of comorbidities, and the cardiovascular reserve of the right ventricle-arterial unit. It can occur suddenly or catastrophic in a previously “healthy heart” or in a hidden way, worsening of compensated RVD in the setting of a chronic heart and lung disease. The diagnosis of acute RVF in ICU patients can become very difficult due to the presence of comorbid conditions that may cause organ hypoperfusion even in the absence of RVD (e.g., sepsis, LV dysfunction, and hypovolemia).

Clinical clues and ECG signs of acute RVD are varied and limited by a low sensitivity and specificity. Therefore, diagnosis typically relies on echocardiography. The ascendance of intensivist-conducted echocardiography has become important not only for early detecting acute RVD in ICU patients but also for monitoring and guiding a rational therapy preventing RVF from occurring.

### 4.1. The Role of Echocardiography

Measurements by two-dimensional echocardiography (2DE) are challenging because of the complex three-dimensional geometry of the RV and sonographic interference from the lungs. While transthoracic echocardiography (TTE) provides adequate imaging in 99% of critically ill patients for diagnosing acute RVD and cardiac cause of shock [62], transesophageal echocardiography (TEE) is adequately suited for identification of ACP and patent foramen ovale [63, 64].

Multiple views are required to an accurate assessment of RV structure and function. We can resume the following views to be used in ICU patients: the parasternal long and short axis, apical four-chamber, and subcostal four-chamber views on TTE and mid-esophageal four-chamber, RV inflow-outflow, and transgastric short axis views on TEE [6, 30, 65, 66].

It is advisable to gather three groups of parameters (Table 2):

- RV structural parameters: linear and areas measurements to assess RV dilation (absolute and relative to LV) predominantly at inlet chamber
- RV functional parameters: predominantly global longitudinal systolic function (since shortening of the RV is greater longitudinally than radially, drawing the tricuspid annulus toward the apex)
- RV afterload assessment.

### 4.2. Pulmonary Artery Catheter (PAC)

Given the potential risks of placing a PAC and the availability of bedside echocardiography, the use of PAC is much less common nowadays. In general, invasive monitoring should be reserved for those patients with echocardiographic evidence of severe RVD at risk of acute RVF or patients with established RVF, since we can perform repeated measurements rapidly [67].

The usual PAC findings suggestive of acute RVD include an elevated CVP (greater than 20 mmHg), an inverse pressure gradient (CVP > PAWP), and a low cardiac index (<2 L/min/m^2^), stroke volume index (<30 mL/m^2^), and mixed-venous oxygen saturation (SvO_2_ < 55%) [68, 69].

One of the challenges of using PAC is the accuracy and precision of PAWP assessment due to the influence of respirophasic effects of mechanical ventilation, end-expiratory versus mean digital measurements, the volume of balloon inflation, and increase extension of zones 1 and 2 (West) [70, 71]. We should be aware that when PEEP is higher than 10 cm H_2_O, PAWP is higher than LV end-diastolic pressure.

In summary, combining the use of real-time echocardiographic evaluation bedside with the knowledge of RV physiology is the desirable way to diagnose acute RVD/RVF in ICU patients. PAC might contribute to the monitoring and adjustment of the treatment.

## 5. Treatment

Effective treatment of acute RVF requires a skilled multidisciplinary team to rapidly assess and triage the patient. The treatment of acute RVD can be divided into the following bundles: (a) general measures including avoiding increasing RV afterload, decreasing RV contractility and optimization of RV preload, applying an “RV-protective” ventilation strategy, and maintaining sinus rhythm and atrioventricular synchrony; (b) pharmacological treatment with a guided inotropic and vasoactive supports; (c) mechanical circulatory support devices. Real-time monitor with bedside echocardiography assessment and the invasive hemodynamic monitoring remain the most valuable methods to guide a rational therapy of acute RVD/RVF in critically ill patients.

### 5.1. General Measures

The prevention of acute RVF in ICU begins with the identification of high-risk patients, for example, patients with severe ARDS and inferior AMI and patients undergoing cardiac surgery with long cardiopulmonary bypass times and receiving cardiac allografts with either long ischemic time or mismatched in size. Once the severe RVD or RVF is recognized, we have to identify and treat any underlying reversible conditions that are either primarily responsible for (triggering factors) or contributing to the progressive impairment of RV function.

Proper management of* volume status* is essential for the failing RV, as both hypovolemia and hypervolemia may result in reduced CO. The RV has a flatter function curve than the LV, meaning that there is less change in RV performance over a wide range of filling pressures. When volume overload is present, the use of diuretics or renal replacement therapy is required [14]. Continuous infusion of diuretics may be preferable over bolus dosing, and the combination of a loop diuretic with a thiazide-like diuretic is indicated whenever diuretic resistance is suspected [72]. Overdiuresis may also be detrimental to RV function, leading to reduced CO, prerenal azotemia, and systemic hypotension.

We should be aware of the limitation of the dynamic fluid responsiveness predictors in fluid management whenever RV dysfunction is present. It is well known that the presence of RV failure should be suspected when a patient has significant variations of stroke volume or pulse pressure but does not respond to fluids [73]. However, the performance of the stroke volume variation and pulse pressure variation could depend on the volume status: during normovolemia their high values failed to predict volume responsiveness (false positive) [74]; by contrast, during hypovolemia their normal values predict volume unresponsiveness (true negative), avoiding dangerous fluid loading [75].

Besides, RV preload requirements differ substantially based on whether afterload is normal or increased. When acute RVD occurs in the setting of increased RV afterload, we should be restrictive with volume management. Increasing blood volume to an already overloaded RV (e.g., PE, ARDS) will not only improve perfusion but also impair CO, aggravating RV dilatation, increasing tricuspid regurgitation and right-sided venous congestion and subsequent underfilling of the LV (ventricular interdependence and serial effect), all of which will lead to hypoperfusion and multiorgan dysfunction. On the contrary, when acute RVD occurs in the setting of normal pulmonary vascular resistance (e.g., RV myocardial infarction), we can be more liberal with fluid reposition to maintain CO. Some authors have proposed a mini-fluid challenge (100 mL of colloid or crystalloid fluid over 1 minute) as a safer and rational approach in some clinical scenarios (e.g., ARDS) [76].

The dominant RV effects of* mechanical ventilation* are to reduce the preload and raise the afterload, which in the setting of acute RVD may be a critical issue. The ventilatory strategy is the main nonpharmacological treatment of the RV afterload through the control of hypoxemia, hypercapnia, acidemia, and inspiratory airway pressure. The main principles of mechanical ventilation for patients with acute RVD include (a) limiting tidal volume and PEEP, therefore limiting plateau (<27 cmH_2_O) and driving pressures (<18 cmH_2_O), (b) avoiding hypercapnia (<60 mmHg) and acidosis, and (c) preventing or reversing hypoxic pulmonary vasoconstriction [30]. Additionally, in ARDS, the presence of RVD (hemodynamic status) and not PaO_2_/FiO_2_ ratio could be an indication for proning to unload the RV by recruiting collapsed alveoli without causing overdistention and reducing airway pressure and hypercapnia (“RV-protective” ventilation strategy) [77–79].

Right atrial contraction contributes up to 40% of RV filling and is more important when the RV compliance is impaired (e.g., RV dilatation). Appropriate sinus heart rate and rhythm, and the maintenance of atrioventricular synchrony and atrial kick, can be among the simplest methods of maintaining and avoiding RV contractility impairment. Electrical or pharmacological cardioversion for the restoration of sinus rhythm and the placement of a temporary pacemaker if heart block is present should be considered [80].

### 5.2. Pharmacological Treatment

The pharmacological treatment will be focused on reducing the RV afterload and preserving an appropriate systemic pressure (vasoactive support) and increasing the RV contractility (inotropes drug therapy). The ideal cardiovascular drug for use in acute RVF would be an agent that enhances systemic arterial pressure and RV contractility without raising pulmonary vascular resistance (PVR). In summary, the pharmacological treatment should provide the following properties: (1) a predominant inotropic property, (2) avoiding pulmonary vasoconstriction, preferably vasodilation, and (3) maintaining systemic perfusion pressure (which is fundamental to RV coronary perfusion) with an adequacy of perfusion (venous oximetry, stroke volume, and CO) [34].

Regarding the* vasopressor support*, the primary objectives are to avoid systemic hypotension, achieving systemic pressure higher than the pulmonary pressure and an optimal PVR/SVR (PVR/systemic vascular resistance) ratio (Table 3). Norepinephrine and a low dose of vasopressin are the preferable drugs (Table 3). Except at high doses, norepinephrine has been shown to increase SVR while reducing pulmonary arterial pressure and PVR/SVR ratio (doses less than 0.5 *μ*g/kg/min) [81]. Norepinephrine is also positively inotropic through the *β*1 adrenergic agonism, increasing cardiac index and improving RV-pulmonary coupling in studies of RVD secondary to PH [14, 82]. Arginine vasopressin (<0.03 U/min) is another vasopressor that preferentially increases SVR over PVR. At higher doses, it should be used with caution since it increases PVR and causes dose-related adverse myocardial effects and coronary vasoconstriction [83]. Phenylephrine improves right coronary perfusion in RVF, although this benefit may be offset by worsening RV function due to increased PVR, and it is not recommended [59, 84].

The next major goal is to improve RV myocardial contractility by using* inotropes*. Dobutamine has favorable pulmonary vascular effects at lower doses (<5 *μ*g/kg/min), although it leads to increased PVR, tachycardia, and systemic hypotension at doses exceeding 10 *μ*g/kg/min [85]. If hypotension occurs, it should be used in combination with vasopressors agents, such as norepinephrine.

Both dopamine and epinephrine are not recommended for tachycardia, arrhythmic events, and an increase in the myocardial oxygen consumption. At moderate-high doses of dopamine, PVR/SVR ratio increases [86] (Table 3).

**Figure 2:**
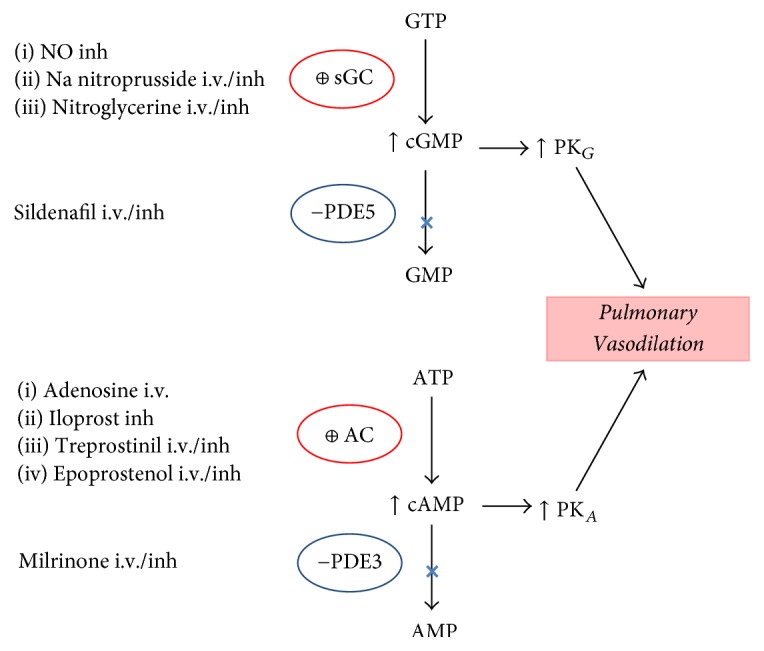
Pulmonary vasodilators drugs, pathways, and mechanisms of action. AC: adenylate cyclase; sGC: soluble guanylate cyclase; ATP and GTP: adenosine and guanosine triphosphate, respectively; cAMP and cGMP: cyclic adenosine and guanosine monophosphate, respectively; inh: inhaled; i.v.: intravenous; NO: nitric oxide; −PDE: phosphodiesterase inhibitor; PK: protein kinase; ⊕: stimulator.

Among* inodilators* (inotropic and vasodilatory properties), both milrinone and levosimendan have been recommended for acute RVD treatment. Milrinone is a bipyridine phosphodiesterase III inhibitor that prevents the degradation of cyclic AMP increasing the intracellular calcium influx such that myocardial contractility improved. Similar to dobutamine, systemic vasodilatation may limit its use. This effect is minimized by the use of inhaled milrinone (Figure 2). Milrinone is usually used in patients with mild-to-moderate RVD undergoing cardiac surgery, but without severe hypotension [25, 87]. Levosimendan is a calcium sensitizer that enhances cardiac contractility without increasing oxygen consumption by increasing calcium sensitivity of cardiomyocyte contractile apparatus during systole, without increasing intracellular calcium concentration, resulting in the acceleration of actin-myosin cross bridge formation rate without prolonging relaxation time (positive lusitropy). It also opens sarcolemma K channels and calcium desensitization in smooth muscle cells, determining vasodilatation in different vascular beds. The opening of mitochondrial inner membrane K_ATP_ channels in cardiomyocytes may be protective for the energy production during ischemia-reperfusion, by preventing mitochondrial calcium overload and preserving high-energy phosphates [88–90]. Among different experimental models, levosimendan improves RV-arterial coupling in acute RVF more than dobutamine [91–93]. We have shown that levosimendan increased RV contractility and improved RV diastolic function and RV-arterial coupling in an experimental model of normotensive PE. This was associated with an improvement of myocardial RV energy status, decreasing the myocardial protein carbonylation [57]. Very recently, in a rodent PE model, we have reported that levosimendan is a more specific vasodilator of resistance PA with a similar relaxant potency to mesenteric arteries, which is preserved after PE but significantly reduced during hypoxia [94]. These novel effects could improve the RV-arterial coupling and preserve an adequate ventilation/perfusion ratio, respectively, during PH treatment. Among clinical scenarios, levosimendan improves RV function and decreases PVR in ischemic RVF and ARDS and after mitral valve replacement surgery [95, 96]. Early perioperative levosimendan treatment in cardiac surgery patients with severely impaired perioperative medical condition appears to reduce mortality and morbidity, and a recent European expert opinion was suggested that the optimal time point for initiation levosimendan (0.1 *μ*g/kg/min) is the day before cardiac surgery [97, 98]. However, very recently, two large, randomized, placebo-controlled trials of levosimendan in patients undergoing cardiac surgery have shown no clear advantage over conventional inotropic drugs for the management of perioperative low cardiac output syndrome [99, 100].

Specific* pulmonary vasodilators* may be useful to reduce RV afterload in acute RVD settings particularly whenever pulmonary remodeling is suspected or confirmed. Exclusion of an isolated pulmonary venous pressure elevation is important, as increased transpulmonary flow may precipitate pulmonary edema [101]. Systemic administration of pulmonary vasodilators may decrease systemic blood pressure, potentially reducing RV preload and worsening RV ischemia. They also can worsen oxygenation by blunting hypoxic pulmonary vasoconstriction and impairing ventilation-perfusion matching. Therefore, the use of inhaled rather than systemic pulmonary vasodilators is strongly recommended [102]. Pulmonary vasodilator therapy relies on three pathways: nitric oxide (NO) donors (guanylate cyclase (GC) stimulators), adenylate cyclase (AC) stimulators, and phosphodiesterase (PDE) inhibitors (Figure 2).

Inhaled NO (iNO) is a potent pulmonary vasodilator at concentrations from 5 to 40 parts per million with a rapid onset of action and very short half-life, making it an ideal agent for management of PH and/or hypoxemia in critically ill patients in whom lowering PAP and improving RV function is paramount (e.g., ARDS, POCS, and heart transplantation) [103–105]. While iNO is the “gold standard” for pulmonary-specific PH treatment, clinicians have been interested in developing less expensive alternatives (Figure 2) [106].

The use of other currently available pulmonary vasodilators, such as the endothelin receptor antagonists (ERA) and the recently approved soluble guanylate cyclase stimulator, riociguat, should probably be avoided in acute RVF due to concerns about unreliable oral absorption. ERA use in the ICU is limited by the potential hepatotoxicity and riociguat may have significant systemic vasodilator effects, especially under conditions such as sepsis. However, oral pulmonary vasodilators can be useful when patients have become hemodynamically stable, and the medical team is planning to withdraw parenteral or inhalation agents, avoiding the rebound of PH [107]. In general, phosphodiesterase type 5 inhibitor (sildenafil) is the preferred agent due to the vast clinical experience [108, 109].

### 5.3. Mechanical Circulatory Support

Despite optimal medical management, some patients fail to improve and require implantation of a mechanical circulatory support device. The RV may exhibit a greater capacity for rapid recovery compared with the LV. Recent literature suggests that 42% to 75% of patients with acute RVF recover hemodynamic and functional status enabling device explantation [110]. The use of extracorporeal life support provides hemodynamic and/or respiratory support in the acute setting, allowing for resolution of a potentially reversible process (bridge to recovery), or bridging who are candidates for transplantation. Options for long-term mechanical circulatory support (destination therapy) are lacking [111, 112]. One of the most important determinants of success is the correct timing of implantation to avoid significant, potentially irreversible end-organ injury [111].

**Figure 3:**
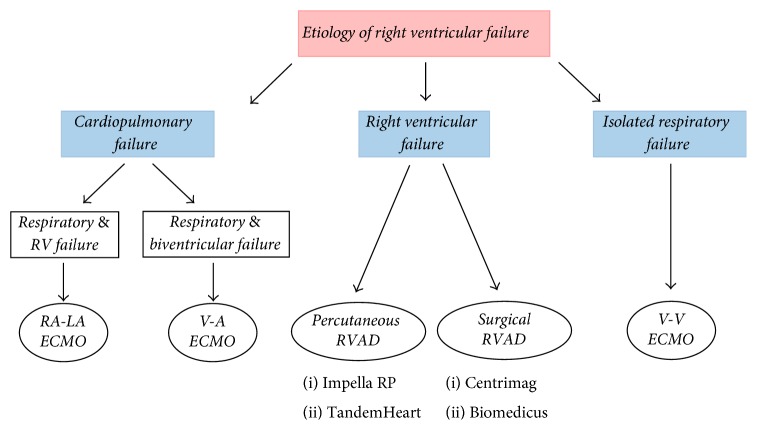
Schematic algorithm for selecting the appropriate extracorporeal life support in patients with refractory right ventricular failure. RA-LA: right atrial-left atrial; RVAD: right ventricular assist device; V-A: venoarterial; V-V: venovenous; ECMO: extracorporeal membrane oxygenation.

Two types of mechanical circulatory assistance have been described in the setting of RVF: (a) RV assist devices (RVAD) and (b) extracorporeal membrane oxygenation (ECMO) [113]. RVAD may be required whenever there is isolated acute RVD/RVF refractory to medical therapy, to sustain the failing RV. All serve to unload and bypass the RV and can be percutaneously (Impella RP®, TandemHeart®) or surgically (Centrimag®, Biomedicus®) implanted [114]. Among the clinical situations to be RVAD considered, we highlight RV myocardial infarction, PE, myocarditis, and postoperative low cardiac output syndrome, following LV assist device implantation or primary graft failure after heart transplantation [66]. Bleeding or thrombus formation is the most common complication related to RVADs [115]. ECMO support with either peripheral or central cannulation is indicated whenever respiratory failure is present while awaiting pulmonary recovery, with or without RVF or biventricular failure. ECMO configuration may be venovenous (VV-ECMO) or venoarterial (VA-ACMO) which present different properties and indications (Table 4) (Figure 3). Infections, the formation of thrombus around the cannulae, and limb hypoperfusion are typical complications of ECMO. Each mechanical circulatory support device should only be used in carefully selected patients (Figure 3).

There was a lack of large comparison groups of patients with RVF managed with medical treatment only, RVADs, or ECMO. A prospective study that includes a clear definition of refractory RVF, guidelines for device use, and appropriate control groups is required.

### 5.4. Targeted Management in Specific Clinical Scenarios

We have described general management considerations for critically ill patients with acute RVF. A key principle in the management of acute RVD focuses on determination and treatment of the underlying etiology [80]. We briefly review targeted therapy for some specific causes of acute RVF (Table 5).

Early myocardial reperfusion of patients with* RV myocardial infarct* (preferably with primary percutaneous coronary intervention) may lead to immediate improvement and later complete recovery of RV function and a better outcome [116]. Unlike the LV, the RV may remain viable for days after an MI [117]. So, late reperfusion is a valid option to consider in patients with acute inferior MI complicated by RVD.

RVF is the principal determinant of early mortality in the acute phase of* pulmonary embolism*. Unless contraindications exist, acute PE is treated with anticoagulation. Based on the contemporary risk classification, “high-risk” patients (persistent arterial hypotension or shock caused by overt RVF) and “intermediate-high-risk” patients (normotensive patients with a high clinical prognostic score plus imaging and biochemical markers of RV function) if RV dysfunction leads to hemodynamic decompensation, reperfusion treatment, preferably systemic (i.v.) thrombolysis, is recommended [3]. Surgical pulmonary embolectomy is an alternative therapy for hemodynamically unstable patients with high-risk PE (particularly if thrombolysis is contraindicated or has failed) and for intermediate high-risk patients in whom hemodynamic decompensation appears imminent, and the bleeding risks of thrombolysis are high [3]. Pharmacomechanical fibrinolysis (catheter-directed fibrinolysis through a multiside hole catheter placed into the thrombus) is another option in these clinical scenarios [118].

Patients with previously unknown* pulmonary arterial hypertension* (PAH) are occasionally seen for the first time in the ICU. Possible triggers for acute RVF in patients with PAH should be actively identified, as their presence will impact clinical management. The most frequent causes are infection/sepsis, supraventricular arrhythmias, anemia with iron deficiency, and nonadherence to or withdrawal from chronic PAH treatment. As we previously mentioned, hypoxia and hypercapnia, as well as acidosis and hypothermia, are precipitating factors of RVF by promoting pulmonary vasoconstriction and the further increase of PAP. Positive pressure ventilation should be avoided because it increases RV afterload and the sedatives should be used with caution because they may lead to systemic hypotension [119]. Fluid status should be closely monitored; if signs of venous and systemic congestion are present, intravenous diuretics should be the first option, followed by renal replacement therapy in patients with diuretic resistance. Parenteral prostanoids are the first-line therapy to achieve a safe reduction of RV afterload. Inhaled pulmonary vasodilators can be used in combination with i.v. therapy to avoid systemic hypotension [107, 120]. In very specific cases, balloon atrial septostomy can be useful to decompresses RV and improve LV filling and CO [121]. It is not recommended in patients with right atrial pressure > 20 mmHg or arterial oxygen saturation < 85% at rest in room air [121].

*Acute respiratory distress syndrome* is the main cause of acute RVF encountered in ICU. Mechanical ventilation can contribute to an uncoupling between pulmonary circulation and the RV, predisposing to the RVF. A protective ventilation strategy with focus on maintaining plateau pressure < 27 cmH_2_O and partial pressure of arterial carbon dioxide < 60 mmHg, adapting positive end-expiratory pressure to RV function, and considering prone positioning for PaO_2_/fraction of inspired oxygen* < *150 mmHg has been recommended to prevent acute RV failure or ameliorate its complications [122].

In* noncardiac surgery*, perioperative RV failure is most often, although not exclusively, secondary to acute pulmonary hypertension (increased afterload). In* cardiac surgery*, RV failure is also frequently caused by volume overload, myocardial ischemia, preexisting RV dysfunction, or arrhythmias [25].

*Right-sided valvular diseases* have a significant and independent impact on morbimortality. Right-sided infective endocarditis accounts for 5–10% of all cases of infective endocarditis and may occur in native valves (intravenous drug abusers), prosthetic valves, congenital heart defects, and implanted devices (e.g., pacemaker) [123]. Surgery is recommended for patients with RVF, severe tricuspid regurgitation, and poor response to diuretics, large vegetation, and recurrent emboli.

## 6. Conclusions

Acute RVD/RVF is seen with increasing frequency in the intensive care unit and causes or aggravates many common critical diseases.

Bedside echocardiography assessment and invasive hemodynamic monitoring remain the most valuable methods to diagnose and to guide a rationale therapy of acute RVD/RVF in critically ill patients.

General precautionary measures, early diagnosis of RVD, and etiology-specific therapy may reduce the appearance of RVF. Supportive therapies focused on improving RV function via optimization of preload, enhancing contractility, and reducing afterload are the key principles in the management of acute RVF.

Future research should focus on better understanding the cellular and molecular mechanisms of acute RV cardiac dysfunction to develop novel therapies that directly target the injured myocardium.

## Figures and Tables

**Table 1:** Acute right ventricular dysfunction definition^*∗*^.

Echo parameters		ECG signs	Biomarkers
RV systolic function	RV dilation		
TAPSE < 16 mm	ED RVD/LVD ratio > 0.9	Complete RBBB	BNP > 100 pg/mL
S < 10 cm/sec	ED RVA/LVA ratio > 0.6	Incomplete RBBB	NT-proBNP > 900 pg/mL
RV fractional area change < 35%	ED RVD > 42 mm (at the base)	Anteroseptal ST elevation	
RV ejection fraction < 45%	ED RVD > 33 mm (at the middle third of RV)	Anteroseptal ST depression	
	Septal dyskinesia in the RV focused view	Anteroseptal T-wave inversion	

BNP: B-type natriuretic peptide; ED RVD/LVD ratio: end-diastolic RV diameter/LV diameter ratio; ED RVA/LVA ratio: end-diastolic RV area/LV area ratio; ED RVD: end-diastole RV diameter; NT-proBNP: N-terminal pro-BNP; S: pulsed Doppler S wave; TAPSE: tricuspid annular plane systolic excursion. ^*∗*^At least one of the items must be present (echo parameters, ECG signs, and biomarkers) [30].

**Table 2:** Cut-off values of RV structural and functional parameters and RV afterload assessment.

RV structural parameters	RV functional parameters	RV afterload assessment
Basal RV diameter^§^ > 42 mm	RV fractional area change ≥ 35%	AccT < 100 msec
RV mid-diameter^§^ > 33 mm	MPI^§^ > 0.43 (pulsed Doppler); >0.54 (tissue Doppler)	Shape of doppler RV outflow tract envelope^#^:
RV EDD/LV EDD^§^ > 0.9	TAPSE^‡^ < 16 mm	(i) No notch
RV/LV EDA^§^ > 0.6	S wave° < 10 cm/s	(ii) Late notch
LV eccentricity index^†^ > 1	Peak RV free wall 2D strain^*∗*^ >−20%	(iii) Midsystolic notch
McConnell's sign^§^		
RV wall thickness > 5 mm		

AccT: acceleration time of RV outflow tract flow; EDD: end-diastolic diameter; EDA: end-diastolic area; LV: left ventricle; RV: right ventricle; MPI: myocardial performance index (the ratio of the sum of isovolumic contraction plus relaxation time and ejection time intervals); S wave: peak velocity of systolic excursion at the lateral tricuspid annulus; TAPSE: tricuspid annular plane systolic excursion. ^#^The presence and position of the systolic notching are related to the pulmonary dynamic afterload severity and RV dysfunction in patients referred for PH [31]. The presence of midsystolic notch is associated with the worst hemodynamic profile. ^§^TTE: apical four-chamber; TEE: mid esophageal four-chamber; ^†^TTE: parasternal midpapillary short axis; TEE: transgastric midpapillary short axis; °TTE: apical four-chamber; TEE: deep transgastric RV; ^*∗*^RV-focused four-chamber view. ^‡^M-mode imaging at the lateral tricuspid valve plane.

**Table 3:** Cardiovascular drugs for the management of acute RVF.

Agent	Receptors agonism					Cardiovascular properties				
	*α*1	*β*1	*β*2	D	V1	CI	PVR	SVR	PVR/SVR	↑ HR
Vasopressors										
*Norepinephrine*	++	+				+	+	++	−/+	+
Phenylephrine	++					−	++	+	+	−
*AVP (0.01–0.03 UI/min)*				+	+	+/−	+/−	++	−	−

Inotropes										
Epinephrine	++	++	+			++	−	++	−	++
Dopamine										
<5 *μ*g/kg/min		+		++		+	−	−	−	+
5–10 *μ*g/kg/min	+	++		++		+	+	+	+/−	+
>10 *μ*g/kg/min	++	++		++		+	+	++	+	+
*Dobutamine*		++	+			++	−	−	−	+

Inodilators										
* Milrinone*						++	−	−	−	+/−
*Levosimendan*						++	−	−	−	+

*α*1, *β*1, and *β*2: adrenergic receptors; D: dopaminergic receptor; V1: vasopressin receptor; +: low-moderate affinity; ++: moderate-high affinity; AVP: arginine vasopressin; CI: cardiac index; PVR and SVR: pulmonary and systemic vascular resistance; HR: heart rate. Drugs in italic are the most preferable. −: neutral effect.

**Table 4:** Differences between venoarterial and venovenous extracorporeal membrane oxygenation (ECMO).

Venoarterial ECMO	Venovenous ECMO
Higher PaO_2_ is achieved	Lower PaO_2_ is achieved
Lower perfusion rates are needed	Higher perfusion rates are needed
Bypasses pulmonary circulation	Maintains pulmonary blood flow
Decreases pulmonary artery pressures	Elevates mixed venous PO_2_
Provides cardiac support to assist systemic circulation	Does not provide cardiac support to assist systemic circulation
Requires arterial cannulation	Requires only venous cannulation

**Table 5:** Mechanisms and targeted management in specific clinical scenarios of acute RV failure.

Clinical scenario	Mechanism	Treatment
Right ventricular infarct	Decreased RV contractility	Early myocardial reperfusion (percutaneous coronary intervention, systemic thrombolysis)
Pulmonary embolism	Increase RV afterload (mechanical obstruction & vasoconstriction)	Systemic anticoagulation, systemic or catheter-directed thrombolysis, embolectomy
Decompensated PAH	Increase RV afterload	Parenteral prostanoids (with or without inhaled pulmonary vasodilators
ARDS	Increasing RV afterload/decreasing RV contractility	Limiting VT and PEEP, avoiding hypoxia, hypercapnia, and acidosis
Noncardiac surgery	Acute PH, decreasing RV contractility (RV infarct)	Pulmonary vasodilators, myocardial reperfusion, inotropic drugs
Cardiac surgery	Volume overload, myocardial ischaemia, preexisting RVD, arrhythmias	Diuretics, inotropic drugs, cardioversion, antiarrhythmic drugs

ARDS: acute respiratory distress syndrome; PAH: pulmonary arterial hypertension; RVD: right ventricular dysfunction.
